# Herniation of the transverse colon into the mediastinum after the use of an omental flap for mediastinitis

**DOI:** 10.1002/ccr3.2481

**Published:** 2019-10-14

**Authors:** Tohru Ishimine, Akio Nakasu, Toshiho Tengan

**Affiliations:** ^1^ Department of Cardiovascular Surgery Okinawa Chubu Hospital Okinawa Japan

**Keywords:** aortic dissection, ascending aortic replacement, herniation of transverse colon, mediastinitis, omental flap

## Abstract

We describe a rare complication, transverse colon herniation into the mediastinum, after the use of an omental flap. Adequate separation of the transverse colon from the omental flap and ensuring that the incision in the diaphragm is as small as possible are important preventive measures.

## Graphical abstract

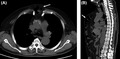

Although the omental flap is often used for mediastinitis after cardiovascular surgery, there are few reports of its associated complications. We present a case with a rare complication after the use of an omental flap. A 71‐year‐old man with a history of hypertension and diabetes mellitus underwent ascending aorta replacement for type A aortic dissection. He developed mediastinitis and underwent sternal debridement with an omental flap 16 days after surgery for aortic dissection. He was discharged 42 days after the surgery for mediastinitis. A swelling along the incision line occurred 3 months postsurgery. CT scan revealed a herniation of the transverse colon into the anterior mediastinum (Figure 1). Although we recommended operation for the herniation, the patient refused the surgery. Apart from swelling of the chest, there have been no symptoms, including pain, during a follow‐up period of 6 years.

**Figure 1:**
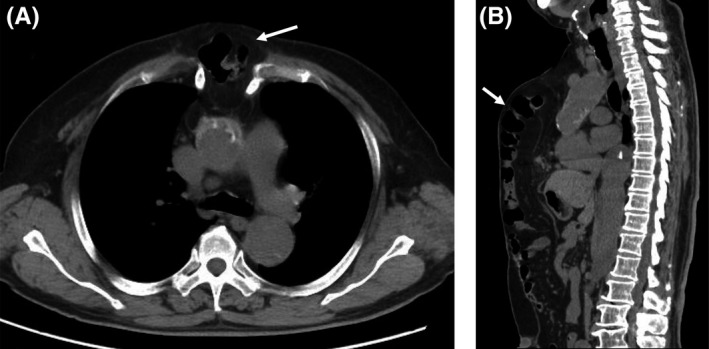
CT scan (A, axial and B, sagittal) showing herniation of the transverse colon into the mediastinum (arrow)

Transverse colon herniation into the mediastinum after the use of an omental flap is rarely reported.^1^, ^2^ Negative pressure of the mediastinum may be a cause of predisposition for organ herniation. Adequate separation of the transverse colon from the omental flap and ensuring that the incision in the diaphragm, through which the omental flap passes, is as small as possible are important preventive measures.

## CONFLICT OF INTEREST

None declared.

## AUTHOR CONTRIBUTIONS

Tohru Ishimine: studied the design, collected the data, and wrote the paper. Toshiho Tengan: reviewed the manuscript. Akio Nakasu: reviewed the manuscript, collected the data, and obtained images.
